# Predictive Equations for Adult Pulmonary Function in Zhejiang Province, China

**DOI:** 10.1155/2022/5500899

**Published:** 2022-03-26

**Authors:** Li Dong, Chengshui Chen, Qi Yang, Yanwen Zheng, Xueren Feng, Fang Chen, Gang Huang, Yuanrong Dai, Zhijie Pan, Huiqing Ge, Tian Zhao, Guangyue Qin, Zhijun Li

**Affiliations:** ^1^The First Affiliated Hospital of Wenzhou Medical University, Department of Respiratory and Critical Care Medicine, Shangcai Village, Nanbaixiang Street, Ouhai District, Wenzhou, Zhejiang Province 325006, China; ^2^The First People's Hospital in Jiaxing, Respiratory Department, 1882 Zhonghuan Nan Road, Nanhu District, Jiaxing, Zhejiang Province 314000, China; ^3^The First People's Hospital in Xiaoshan, Respiratory Department, 199 Shixin Middle Road, Xiaoshan District, Hangzhou, Zhejiang Province 311201, China; ^4^The Central Hospital in Huzhou, Department of Respiratory and Critical Care Medicine, 198 Hongqi Road, Wuxing District, Huzhou, Zhejiang Province 313000, China; ^5^The TCM Hospital of Zhejiang Province, Department of Pulmonary Function Examination, 54 Youdian Road, Shangcheng District, Hangzhou, Zhejiang Province 310000, China; ^6^The Second Affiliated Hospital of Zhejiang University, Department of Respiratory and Critical Care Medicine, 88 Jiefang Road, Shangcheng District, HangZhou, Zhejiang Province 310009, China; ^7^The Second Affiliated Hospital of Wenzhou Medical University, Department of Respiratory and Critical Care Medicine, 109 Xueyuan West Road, Lucheng District, Wenzhou, Zhejiang Province 325027, China; ^8^The First Affiliated Hospital of Zhejiang University, Department of Respiratory and Critical Care Medicine, 79 Qingchun Road, Shangcheng District, HangZhou, Zhejiang Province 310009, China; ^9^The Run Shaw Hospital of Zhejiang University, Department of Respiratory and Critical Care Medicine, 3 Qingchun East Road, Shangcheng District, HangZhou, Zhejiang Province 310036, China; ^10^Zhejiang Hospital, Department of Respiratory Medicine, 1229 Gudun Road, Xihu District, Hangzhou, Zhejiang Province 310000, China

## Abstract

**Background:**

Accurate interpretation of lung function tests requires appropriate spirometry reference values derived from large-scale population-specific epidemiological surveys. The aim of this cross-sectional study was to establish normal spirometric values for the population of healthy, nonsmoking Han Chinese adults residing in Zhejiang province, China.

**Methods:**

We measured lung function parameters such as forced expiratory volume in 1 s, forced vital capacity, peak expiratory flow, maximal midexpiratory flow, and diffusion capacity for carbon monoxide and considered age, height, and weight as independent factors that may modify these parameters. The clinical data were divided into the study arm and validation group. The study arms were used to construct predictive equations using stepwise multiple linear regression, and data from the validation group were used to assess the robustness of the equations.

**Results:**

The 3866 participants were randomized into a study arm (*n* = 1,949) and a validation arm (*n* = 1,917). Lung function parameters had a negative association with age and a positive association with height. Data from the two groups were similar. Predictive equations were constructed from the study arm, and the validation group was used to test the feasibility of the reference equations.

**Conclusions:**

The reference values we derived can be used to evaluate lung function in this cohort in both epidemiological studies and clinical practice.

## 1. Introduction

Spirometry is a valuable evaluation tool and is used extensively as an objective measure of lung function for a variety of purposes, such as diagnosing respiratory disease, quantifying disease severity, and assessing prognosis [1]. Lung function tests are also an important part of sports medicine and occupational health. However, accurate interpretation of lung function tests requires appropriate reference values derived from epidemiological surveys of relevant populations with local and ethnic specificity.

Most published predictive equations from spirometry are derived from data from European and American populations after adjustment for ethnicity as a factor [2]. However, Jian et al. [3] reported that Caucasian reference values are not suitable for Chinese populations in any age range, even after correction with fixed ethnic conversion factors. Thus, the researchers established new reference values, for Chinese people aged 4–80 years [3]. However, Jian et al.' [3] study examined individuals from only one hospital in Zhejiang province and included a large number of participants under 18 years of age. Other studies reported spirometric reference values for Chinese populations, but were limited by small samples, restricted age ranges, or the locality of the sampled population [4, 5]. Moreover, the findings of these previous studies demonstrated that regional disparities still exist within a given ethnic group. Therefore, it is necessary to establish appropriate and specific pulmonary function reference values in given population.

A variety of factors can affect lung function, such as age, sex, height, weight, chronic exposure to harmful chemicals, and air pollution, such as PM 2.5. In addition, ethnicity, regional environment, and economic development level have also been reported to have an impact on pulmonary function [6–12]. Weitz et al. [13] reported that lung function values in the Chinese population are also affected by altitude. The population of Zhejiang province, which is located in the eastern coastal area of China, is primarily composed of Han Chinese. Zhejiang is well-developed economically and has various unique lifestyle characteristics and customs that differ from those in other regions in China. Consequently, there is strong demand for pulmonary reference values for the adult population in Zhejiang province.

On the basis of previous research, the present study was conducted to establish spirometry reference values for the healthy adult population using a multicenter large-sample design and to validate predictive equations in Zhejiang province.

## 2. Materials and Methods

### 2.1. Study Design

This cross-sectional study was conducted with a uniform study protocol between March 2018 and March 2020 in 11 regions with 33 collaborative centers across Zhejiang province. There were at least three collaborative centers in each region. The study was conducted according to the World Medical Association Declaration of Helsinki in 1975, as revised in 1983, and was approved by the Ethics Committee of Zhejiang Hospital. Written informed consent was obtained from all participants.

### 2.2. Estimated Sample Size

A stratified multistage cluster random sampling method was used to determine an appropriate sample size in the present study. Detailed information is provided in the Supplementary Materials.

### 2.3. Participants

All participants were recruited at the pulmonary function department for health screenings of each center. The main inclusion criteria were as follows: aged 18–80 years; never-smoker; body mass index between 18.5 and 23.9 kg/m^2^; no known exposure to harmful gases, dust, or high levels of air pollution; longtime resident of Zhejiang province; no symptoms or history of chronic cardiopulmonary diseases, such as chronic bronchitis, asthma, lung cancer, pulmonary fibrosis, pulmonary tuberculosis, chronic heart diseases, or chronic obstructive pulmonary disease; and no respiratory tract infection within the preceding 4 weeks. Age was determined by date of birth. Anthropometrical indices such as height and weight were also recorded.

### 2.4. Spirometry

At each collaborative center, pulmonary function was measured using spirometry (Jaeger, German) in accordance with the American Thoracic Society recommendations for spirometry [14]. Technicians were trained by video according to the procedures and criteria of the American Thoracic Society and European Respiratory Society [15]. The details are listed in the Supplementary Materials.

The process was repeated for at least three replicates to obtain repeatable spirograms. Acceptable differences were within 150 mL between the two best sets of results. The highest FVC, forced expiratory volume in 1 s (FEV_1_), and peak expiratory flow (PEF) values were selected from the best sets of results, while the maximal midexpiratory flow (MMEF) was taken from the maneuver with the largest sum of FVC and FEV_1_. Other parameters, such as vital capacity, tidal volume, inspiratory reserve volume, and expiratory reserve volume, were also collected from the best measurement. A helium and carbon monoxide gas analyzer was used to detect the lung diffusion capacity for carbon monoxide (DLCO) and dispersion rate. Single-breath diffusion was performed at least twice to measure the diffusion function. Spirometry was performed between 8:00 and 16:30.

### 2.5. Data Collection

Case report forms were designed to obtain detailed information, and original spirometry reports were submitted to Zhejiang Hospital for quality control after preliminary screening at the originating center. Quality control was conducted at Zhejiang Hospital by two independent specialists (Tian Zhao and Guangyue Qin) according to the criteria of the American Thoracic Society and European Respiratory Society [14, 15]. Disagreements were resolved by a senior physician. The authors had access to information that could identify individual participants during or after data collection. All data were double-entered and checked using EpiData 3.0 (Odense, Denmark).

### 2.6. Statistical Analysis

We used SPSS version 19.0 for statistical analysis (IBM SPSS, Armonk, NY, USA). Participants were randomly divided into a study group and a validation group, and measurement data were presented as mean ± standard deviation. The Kolmogorov–Smirnov test was used to assess the normality of data distributions. Pearson's correlations were used to calculate the associations between age, height, and weight and lung function parameters in each subgroup. The predictive equations for lung function parameters (FVC, FEV_1_, FEV_1_/FVC ratio, PEF, MMEF, and DLCO) were deduced from study groups using stepwise multiple linear regression, and age, height, and weight were regarded as independent variables. The predicted mean percentage values in the validation group were compared with those in the study group using *t*-tests to validate the efficiency of equations. A level of *p* < 0.05 was considered to indicate statistical significance.

## 3. Results

### 3.1. Participants

**Figure 1:**
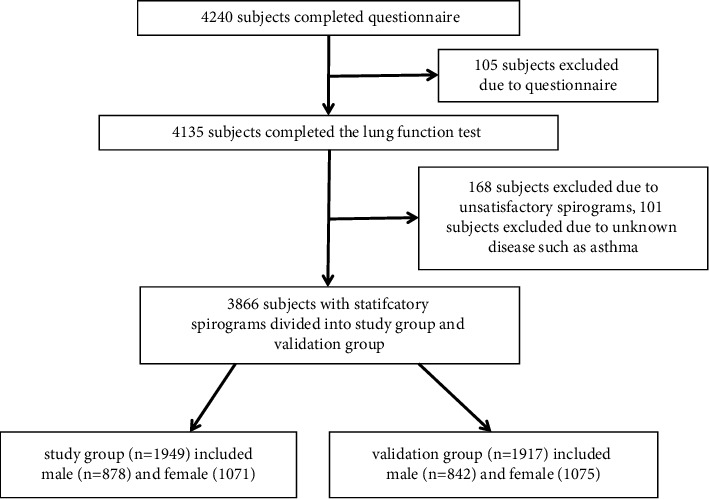
Flowchart of subjects included into the study.

**Figure 2:**
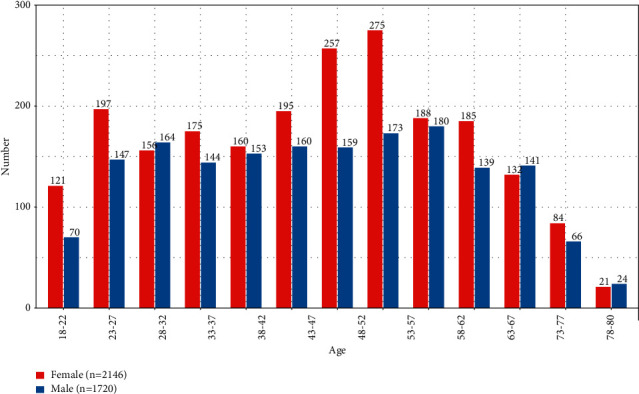
The age distribution of the sample.

In total, 33 collaborative centers and 4,456 participants were recruited for the study. After preliminary screening of questionnaires at each center, a total of 3,866 individuals (1720 men and 2146 women) provided spirometry measurements (Figure 1). The age distribution of the samples is shown in Figure 2. The participants were divided into a study population (*n* = 1,949) and a validation population (*n* = 1917) by randomization (Table 1). The basic characteristics of both groups are given in Table 2. No significant differences were identified between the groups for any parameters.

### 3.2. Pearson's Correlation Analysis

Pearson's correlation was performed to calculate the associations between age, height, and weight and lung function parameters in each subgroup according to sex (Table 3, Supplementary Figure 1). In both men and women, a significant negative association was observed between lung function parameters and age, and height had a significant positive association with spirometry parameters except for FEV1/FVC. In addition, the results suggested that weight had a positive association with FVC, FEV1, PEF, MMEF, and DLCO, but a negative association with FEV1/FVC in the male subgroup. In the female subgroup, positive associations were also identified between weight and FVC, FEV1, PEF, and DLCO, whereas negative associations were identified between weight and FEV1/FVC and MMEF.

### 3.3. Construction and Validation of Predictive Equations

Stepwise multiple regression was performed to construct predictive equations based on lung function parameters as dependent variables and height, weight, and age as independent variables (Table 4). The raw and adjusted *R*^2^ values were almost identical and indicated that spirometry parameters, such as FEV1, FVC, FEV1/FVC, MMEF, and DLCO, were negatively associated with age in both the male and the female groups. Height had a positive association with spirometry parameters, except for FEV1/FVC in the female group. In addition, weight had a positive association with several parameters, especially for FEV1/FVC and DLCO in the male group and FVC, PEF, and DLCO in the female group. A verification sample was used to validate the utility of the predictive equations, and no significant difference was identified (Table 5). Most lung function parameters were higher in men, with the exception of the FEV_1_/FVC ratio and PEF, which were higher in women. Similar values were obtained for the study and validation groups, to which participants had been randomly assigned, confirming the validity of the reference equations.

## 4. Discussion

The present study was designed to identify normal lung function values in healthy, nonsmoking adults from Zhejiang province using predictive equations derived from spirometry data. Age, weight, and height were included in the model because they are known to influence lung function. We validated the predictive equations by randomizing the participant cohort into two groups and comparing their data, which were similar. The results suggested that most of the healthy participants could be identified by FEV1 or FVC values above 80% of the predicted values using these equations.

Lung function can be influenced by multiple factors, including gender, age, height, and weight, so predictive values depend on these variables. Males and females in our study differed significantly in many characteristics, and males exhibited significantly higher FEV1%pred, FVC%pred, MMEF%pred, and DLCO%pred, with the exception of FEV1/FVC%pred and PEF%pred. Age has historically been considered a major factor in lung function. In healthy participants, lung function increases throughout the growth stage, peaks around the young period [16], declines gradually because of increased rigidity of the chest wall, decreases in expiratory muscle strength, and dysfunction of the smaller airways. Various indices of lung function, including DLCO, decline with age [17] because gas exchange tends to decrease following loss of alveolar surface area and reduced blood volume. Similarly, the current study recruited subjects aged 18–80 years, certified that lung function parameters (FVC, FEV1, FEV1/FVC, PEF, MMEF, and DLCO) were negatively associated with age, which had accordance with the previous research [17]. In future, a stratified analysis according to age should be performed to clarify the impact of age on spirometry parameters.

Height, as the second strongest determining factor, was also used to establish lung function predictive equations. A study conducted by Quanjer et al. demonstrated that, in terms of FEV1, FVC, and FEV1/FVC, standing height was used to evaluate lung function and led to low percentages of misclassifications in the clinic [18]. Gao et al. [19] reported that the variance of spirometry parameters increased with increasing height, suggesting that, in taller individuals, the predictive values for lung function involved in FVC, FEV1, PEF, and MMEF are larger, whereas decreasing lung volume was observed in shorter participants. In addition, we found a positive association between height and FEV1, FVC, FEV1/FVC, PEF, and MMEF in the present study. A previous study reported that DLCO was positively correlated with height [20], and this was also confirmed in our Zhejiang cohort. In addition, the current findings indicated that height had a positive impact on DLCO.

The impact of bodyweight on pulmonary function has been examined in many previous studies. Contradictory conclusions have been reported in the literature regarding FEV_1_ and FVC. One study reported that weight decreased these parameters [21]. In addition, McCallister et al. reported that obesity can cause a parallel reduction in FEV1 and FVC because of airway limitations and then preserve the FEV1/FVC ratio constant [22]. In addition, a study conducted by Peralta et al. also reported that there was no association between weight change profiles and FEV1/FVC decline [23]. However, it remains unclear whether weight affects DLCO. A negative association with body mass index is possible, but has not been confirmed [24]. Therefore, the role of weight as a factor in lung function is unknown. Different from the previous research, we found weak to moderate associations between weight and FEV1/FVC and DLCO in men and FVC, PEF, and DLCO in women in the present study, which suggested that weight contributes to oxygen diffusion.

### 4.1. Limitations

We did not measure exogenous factors that can affect pulmonary function, such as levels of air pollution, and did not survey participants regarding diet and exercise. In addition, we did not compare the results of pulmonary function tests with measurements taken after the use of a bronchodilator. This measure should be included in the protocol in a future study to validate the current results.

## 5. Conclusion

We derived values for normal lung function in healthy adult Han Chinese participants from a large and representative population sample in Zhejiang province. These new reference values can be used to evaluate lung function in this cohort in both epidemiological studies and clinical practice.

## Figures and Tables

**Table 1:** Participants' basic characteristics.

Group	*N*	Gender		Age	Height	Weight
		Male	Female			
Study group	1949	878	1071	47.80 ± 15.84	163.51 ± 8.00	61.06 ± 9.71
Validation group	1917	842	1075	47.69 ± 15.79	163.06 ± 7.83	60.82 ± 9.51

**Table 2:** Participants' mean clinical data.

	Male		Female	
	Study group (878)	Validation group (842)	Study group (1071)	Validation group (1075)
Age	47.77 ± 15.76	48.13 ± 16.17	47.82 ± 15.92	47.35 ± 15.49
Height	169.6 ± 6.3	169 ± 6.36	158.52 ± 5.34	158.4 ± 5.32
Weight	67.33 ± 8.8	66.6 ± 8.78	55.92 ± 7.04	56.29 ± 7.37
VC	4.08 ± 0.77	4.04 ± 0.83	2.94 ± 0.56	2.96 ± 0.56
FRV	3.39 ± 0.83	3.33 ± 0.82	2.88 ± 1.61	2.8 ± 0.55
TLC	5.36 ± 1.15	5.38 ± 1.13	4.69 ± 0.75	4.68 ± 0.71
RV	1.94 ± 0.63	1.94 ± 0.62	1.76 ± 0.51	1.72 ± 0.48
RV/TLC	35.63 ± 9.29	35.43 ± 8.93	38.87 ± 10.26	38 ± 8.96
VT	1.17 ± 0.6	1.17 ± 0.65	0.94 ± 0.51	0.93 ± 0.46
IC	2.86 ± 0.69	2.8 ± 0.63	2.09 ± 0.46	2.11 ± 0.46
IRV	1.53 ± 0.27	1.52 ± 0.23	1.38 ± 0.21	1.39 ± 0.21
ERV	1.26 ± 0.67	1.24 ± 0.68	1.17 ± 1.04	1.14 ± 0.2
FVC	3.88 ± 0.73	3.83 ± 0.78	2.78 ± 0.55	2.79 ± 0.55
FEV1	3.42 ± 0.69	3.38 ± 0.73	2.47 ± 0.53	2.47 ± 0.54
FEV1%	83.47 ± 6.44	83.72 ± 6.65	85.37 ± 16.06	84.81 ± 6.33
PEF	8.52 ± 1.89	8.48 ± 1.92	5.94 ± 1.36	6.04 ± 1.38
MMEF	4.76 ± 1.36	4.73 ± 1.39	3.48 ± 1.04	3.57 ± 1.07
FEF25%	6.49 ± 1.56	6.48 ± 1.64	4.77 ± 1.18	4.87 ± 1.18
FEF50%	3.45 ± 1.14	3.42 ± 1.15	2.71 ± 1.68	2.71 ± 0.89
FEF75%	1.58 ± 0.88	1.55 ± 0.8	1.23 ± 0.68	1.22 ± 0.66
MVV	127.97 ± 26.7	126.44 ± 27.51	106.38 ± 13.76	107.31 ± 16.29
DLCo	9.88 ± 4.68	9.78 ± 4.61	8.03 ± 4.35	7.93 ± 4.08
Specific diffusion	1.93 ± 1.31	1.92 ± 1.28	2.09 ± 1.43	2.07 ± 1.39
VA	5.56 ± 0.85	5.49 ± 0.82	4.6 ± 0.54	4.58 ± 0.52

**Table 3:** Pearson's correlation analysis of lung function parameters and age, height, and weight, stratified by sex.

Gender		FVC	FEV1	FEV1/FVC	PEF	MMEF	DLCO
Male	Age	−0.664 ^*∗∗*^	−0.714 ^*∗∗*^	−0.387 ^*∗∗*^	−0.385 ^*∗∗*^	−0.515 ^*∗∗*^	−0.180 ^*∗∗*^
	Height	0.590 ^*∗∗*^	0.574 ^*∗∗*^	0.115 ^*∗∗*^	0.380 ^*∗∗*^	0.351 ^*∗∗*^	0.154 ^*∗∗*^
	Weight	0.307 ^*∗∗*^	0.257 ^*∗∗*^	−0.085 ^ ^*∗*^^	0.210 ^*∗∗*^	0.132 ^*∗∗*^	0.145 ^*∗∗*^

Female	Age	−0.628 ^*∗∗*^	−0.691 ^*∗∗*^	−0.149 ^*∗∗*^	−0.374 ^*∗∗*^	−0.545 ^*∗∗*^	−0.127 ^*∗∗*^
	Height	0.582 ^*∗∗*^	0.552 ^*∗∗*^	0.028	0.326 ^*∗∗*^	0.316 ^*∗∗*^	0.154 ^*∗∗*^
	Weight	0.129 ^*∗∗*^	0.071 ^*∗*^	−0.053	0.123 ^*∗∗*^	−0.014	0.102 ^*∗∗*^

^*∗∗*^*P* < 0.01,  ^*∗*^*P* < 0.05.

**Table 4:** Predictive equations of lung function.

	Regression equations	*R* ^2^	Adjusted *R*^2^
Male			
FVC	−2.929–0.024 ^*∗*^A + 0.047 ^*∗*^H	0.583	0.582
FEV1	−2.127–0.026 ^*∗*^A + 0.04 ^*∗*^H	0.626	0.625
FEV1/FVC	85.76–0.156 ^*∗*^A-0.132 ^*∗*^H + 0.083 ^*∗*^W	0.173	0.170
PEF	−3.994−0.034 ^*∗*^A + 0.083 ^*∗*^ H	0.216	0.214
MMEF	−0.366–0.039 ^*∗*^A + 0.041 ^*∗*^ H	0.297	0.296
DLCO	3.116–0.045 ^*∗*^A + 0.031 ^*∗*^H + 0.054 ^*∗*^W	0.049	0.045
Female			
FVC	−2.913–0.017 ^*∗*^A + 0.039 ^*∗*^H + 0.005 ^*∗*^W	0.548	0.547
FEV1	−2.192–0.019 ^*∗*^A + 0.035 ^*∗*^H	0.589	0.588
FEV1/FVC	92.573–0.151 ^*∗*^A	0.022	0.021
PEF	−0.799–0.028 ^*∗*^A + 0.044 ^*∗*^H + 0.021 ^*∗*^W	0.193	0.191
MMEF	0.547–0.032 ^*∗*^A + 0.028 ^*∗*^H	0.315	0.314
DLCO	−4.343–0.03 ^*∗*^A + 0.055 ^*∗*^H + 0.068 ^*∗*^W	0.036	0.033

A, age; H, height; W, weight.

**Table 5:** Normal lung function values calculated for adults in Zhejiang province.

Gender	Group	FVC	FEV1	FEV1/FVC	PEF	MMEF	DLCO
Male	Study group	101.83 ± 0.13	101.32 ± 0.13	74.05 ± 0.05	104.19 ± 0.26	109.95 ± 1.25	111.93 ± 0.33
	Validation group	102.88 ± 0.16	102.13 ± 0.16	73.82 ± 0.06	103.87 ± 0.26	107.14 ± 0.39	113.55 ± 0.45

Female	Study group	100.49 ± 0.16	101.28 ± 0.27	100.95 ± 0.08	106.51 ± 0.28	107.22 ± 0.37	96.62 ± 0.28
	Validation group	100.3 ± 0.15	100.89 ± 0.15	101.21 ± 0.07	105.49 ± 0.31	104.42 ± 0.32	97.47 ± 0.33

## Data Availability

The data used to support this study are stored in Respiratory Department in Zhejiang Hospital and are available from the corresponding author upon request.

## Supplementary Materials

Supplementary Materials The supplementary materials included estimated samples size and pulmonary function test method. In addition, the Supplementary Figure 1 shows lung function parameters and age, height, and weight both female and male.
