# Supplementary material for: Predictive Equations for Adult Pulmonary Function in Zhejiang Province, China
Source: J Trop Med. 2022 Mar 26;2022:5500899. doi: 10.1155/2022/5500899 (PMC8976640; doi:10.1155/2022/5500899)
Supplement: Supplementary Materials — The supplementary materials included estimated samples size and pulmonary function test method. In addition, the Supplementary Figure 1 shows lung function parameters and age, height, and weight both female and male. [file 5500899.f1.doc]

**Supplementary Materials**

***Estimated for samples size***

Stratified multi-stage cluster random sampling method was engaged in the study. First of all, the sample size under simple random sampling was estimated to be *n*, forced vital capacity (FVC) with (3.5 ± 0.9) was selected as the main endpoint to estimate the sample size. According to the sample size calculation formula of the overall mean of the measurement data, *n* was calculated as 385. In the light of the gender, urban and rural stratification factor *str*, the loss rate (10%), and *deff* (2.5), the total sample size was n = *deff* * *n* * *str* * 1.1 = 2.5 * 385 * (2 * 2) * 1.1 = 4235. Finally, the sample size was determined to be 4290 based on the convenience of distribution.

***Spirometry***

At each collaborative centers, pulmonary function was measured using spirometry (Jaeger, German) met the American Thoracic Society recommendations for spirometry (Miller et al. 2005). Technicians were trained by video according to the procedures and criteria of the American Thoracic Society and European Respiratory Society (Pellegrino et al. 2005). Prior to measurements, a 3-L cylinder was used for calibration checks and a difference within 3% between the measured value and the theoretical value was regarded as reasonable. Calibration reports were printed and filed. Environmental calibration was also performed according to operational standards. The selected posture involved sitting straight and maintaining the head at a natural level. Subjects were instructed to attach a nose clip or perform manual occlusion of the nostrils, place the mouthpiece in the mouth, and close the lips around the mouthpiece. Subjects were then encouraged to relax their breathing for measurement of static parameters for at least 10 cycles, and to exhale maximally until no air could be expelled, then inhale completely while maintaining an upright posture. Finally, subjects were asked to inhale maximally and rapidly to total lung capacity with a pause of <1 s. Acceptable maneuvers included no early termination or cut-off during exhalation or inspiration, maximal effort, no gas leakage or obstruction of the mouthpiece, extrapolated volume <5% of FVC or 0.15 L, and a volume-time curve that reached a plateau or forced expiratory time ≥6 s in subjects.


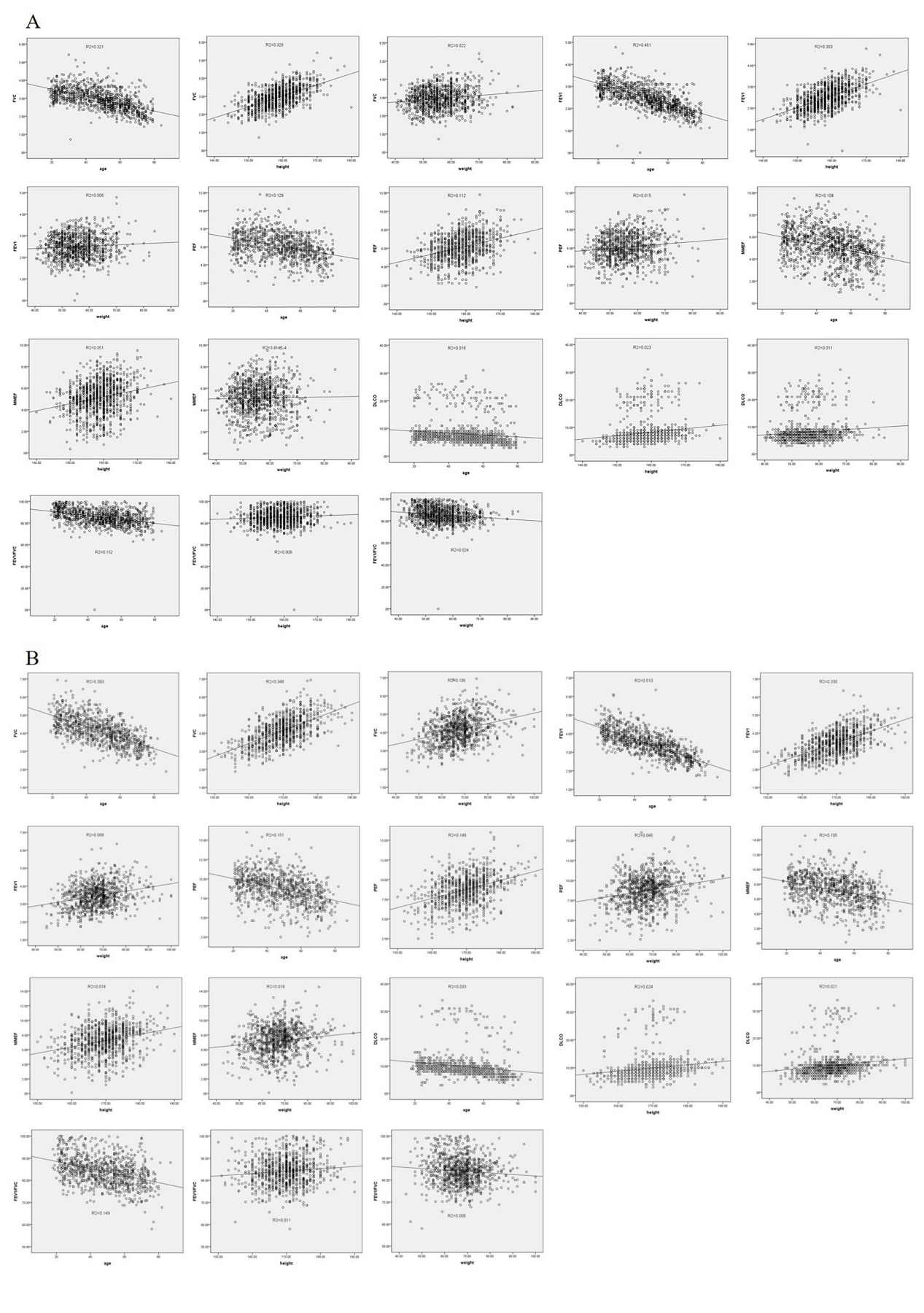


Fig 1: The associations between lung function parameters and age, height and weight in each subgroups based on gender (A: Female; B: Male)
